# Striatal Interneurons in Transgenic Nonhuman Primate Model of Huntington’s Disease

**DOI:** 10.1038/s41598-019-40165-w

**Published:** 2019-03-05

**Authors:** Shoeb B. Lallani, Rosa M. Villalba, Yiju Chen, Yoland Smith, Anthony W. S. Chan

**Affiliations:** 1 Department of Human Genetics, Emory University School of Medicine, Atlanta, GA, USA; 2 Department of Neurology, Emory University School of Medicine, Atlanta, GA, USA; 3 Division of Neuropharmacology and Neurological Diseases, Yerkes National Primate Research Center, Atlanta, GA USA; 4 UDALL Center for Excellence for Parkinson’s Disease, Emory University, Atlanta, GA USA

## Abstract

Huntington’s disease is an autosomal dominant neurodegenerative disorder associated with progressive motor and cognitive impairments, and the expansion of a cysteine-adenine-guanine trinucleotide (polyglutamine) repeats in exon one of the human huntingtin gene. The pathology of the disease is characterized by a profound degeneration of striatal GABAergic projection neurons with relative sparing of interneurons accompanied with astrogliosis. Here, we describe the striatal pathology in two genotypically different transgenic HD monkeys that exhibit degrees of disease progression that resembled those seen in juvenile- (rHD1) and adult-onset (rHD7) HD. The caudate nucleus and putamen underwent severe neuronal loss in both animals, while the striatal volume was reduced only in rHD1, the most severely affected monkey. The number of GABAergic (calretinin- and parvalbumin-positive) and cholinergic interneurons was also reduced in most striatal regions of these two monkeys, but to variable degrees. Overall, the density of interneurons was increased in rHD1, but not in rHD7, suggesting a relative sparing of interneurons over projection neurons in the striatum of the most affected HD monkey. The neuropil of both the caudate nucleus and putamen was invaded with reactive astrocytes in rHD1, while astrogliosis was much less severe in rHD7 and absent from control. Combined with behavioral data collected from these monkeys, our findings further demonstrate that transgenic HD monkeys share similar disease patterns with HD patients, making them a highly reliable preclinical HD animal model.

## Introduction

Huntington’s disease (HD) is an aggressive autosomal dominant neurodegenerative disorder that is caused by a polyglutamine expansion (polyQ > 36) in exon 1 of the human huntingtin (*HTT*) gene^1,2^. Currently, there is no cure for HD, making the need for an animal model that can effectively recapitulate progressive behavioral decline and neuroanatomical changes of HD an important factor for advancing novel therapeutic development. Unlike most rodent models, transgenic HD monkeys exhibit progressive development of motor, cognitive, and neuropathological dysfunctions similar to those observed in HD patients^3,4^. Thus, the continued development and characterization of a monkey model of HD is important for elucidating disease etiology, pathogenesis, and for the development of cures^3,5–9^.

HD is clinically characterized by a major decrease of striatal volume and enlarged lateral ventricles. These changes are associated with the degeneration of striatal projection neurons, whereas various populations of GABAergic and cholinergic interneurons are thought to be relatively spared (Cicchetti *et al*., 2000). However, it is noteworthy that these conclusions were largely based on interneurons density measurements, instead of absolute counts of specific populations of interneurons. Thus, because the striatal volume of advanced HD patients is dramatically reduced^10–12^, these data do not provide information about the degree of interneurons loss in HD. In addition to neuronal pathology, severe astrogliosis was also reported in degenerated striatal regions of advanced HD patients^11^.

The present study reports data about interneuron counts and astrogliosis in the caudate nucleus and putamen of two HD monkeys (rHD1 and rHD7) and one age-matched control animal (rWT1). HD monkeys expressing exon one with 29Q of the human *HTT* gene regulated by the human polyubiquitin C promoter (rHD1) or expressing exons 1–10 with ~70Q of the human *HTT* gene regulated by the human *HTT* promoter (rHD7) were used in this study. Behavioral and longitudinal MRI analysis of these monkeys showed that they bare a strong resemblance to behavioral and anatomical changes observed in human HD patients, suggesting that this monkey model may recapitulate clinical changes associated with HD^3,4,7^. To further validate the striatal pathogenesis of these HD monkeys, we used an unbiased stereological method to assess the loss of specific subtypes of GABAergic and cholinergic interneurons in various functional regions of the caudate nucleus and putamen. The extent of reactive astrocytes throughout the striatum of these animals was also determined.

## Material and Methods

### Ethics Statement

All animal procedures were approved by the Institutional Animal Care and Use Committee (IACUC) and the Biosafety Committee of Emory University, and were performed according to the Guide for the Care and Use of Laboratory Animals and the U.S. Public Health Service Policy on the Humane Care and Use of Laboratory Animals.

### Animals

For stereological analysis, two HD monkeys and one control animal were used. The two HD monkeys, rHD1 and rHD7, were 5 years old at the time of euthanasia. rHD1 was a five-year-old male transgenic HD-monkey who was generated via lentiviral transfer of exon 1 of the mutant *HTT* transgene with 29Q repeats regulated by human ubiquitin-C promoter and a green fluorescent protein (GFP) regulated by human ubiquitin-C promoter for visualization. rHD7 was a five-year-old male transgenic HD-monkey that was generated via a lentiviral transfer of exons 1–10 of the mutant *HTT* gene with 70Q repeats regulated by a human *HTT* promoter. rWT1 was a four-year-old male monkey who served as the control. Longitudinal behavioral and neuroimaging data about these two HD monkeys have been recently published^3,4^. Because of the distinct genotypes, rHD1 displayed a more aggressive form of HD than rHD7.

### Tissue Preparation

The monkeys were deeply anesthetized via pentobarbital overdose (100 mg/kg) at the time of euthanasia. rWT1 was transcardially perfused with a mixture of paraformaldehyde (4% PFA) and glutaraldehyde (0.1%). In the cases of rHD1 and rHD7 monkeys, they were flushed transcardially with cold PBS. After perfusion, the brains were taken out from the skull and post-fixed in 4% paraformaldehyde (48 hours for rWT1 and 2 weeks for rHD1 and rHD7). In the cases of rHD1 and rHD7, only one hemisphere was post-fixed and used for the present study. The other hemisphere was dissected into constituent regions, cut into smaller pieces, snap frozen in liquid nitrogen, and stored in −80 °C for future biochemical analyzes. After fixation, the brains were washed with PBS, saturated in 30% sucrose, serial frozen-sectioned into 50 µm thick coronal slices and stored in an anti-freeze solution (1.4% NaH_2_PO_4_-H_2_O, 2.6% Na_2_HPO_4_, 30% ethylene glycol, 30% glycerol in H_2_O) at −20 °C.

### Immunohistochemistry Experiments

Coronal sections that covered the full rostrocaudal extent of the striatum in the two HD (rHD1, rHD7) and the control (rWT1) monkey were used in these experiments. To test for possible regional differences in the extent of striatal degeneration, sections were divided into pre-commissural and post-commissural levels using the anterior commissure as landmark between the two regions.

Nissl staining was performed on 1 every 12 sections in each monkey. Adjacent serial sections were immunostained with specific antibodies for calretinin (CR), parvalbumin (PV) (SWANT, Switzerland) or choline acetyltransferase (ChAT) (PROSCI, Poway, CA, USA) to localize specific populations of striatal interneurons according to the order provided in Supplemental Table 1. Another series of adjacent sections was immunostained with an antibody against Glial Fibrillary Astrocytic Protein (GFAP, MILLIPORE-SIGMA, Burlington, MA, USA). The specificity of these antibodies has been characterized in previous studies^13,14^ and by the manufacturers. Overall, the pattern of immunostaining obtained with these antibodies was consistent with areas known to express detectable levels of mRNA encoding for these proteins and the distribution of labeling published in other studies using the same or different antibodies^15–21^.

The immunostaining protocol was the same for each antibody. In brief, single striatal sections were treated at room temperature (RT) with sodium borohydride for 20 min. followed by a pre-incubation for 1 hour (h) in a solution containing 1% normal horse serum, 0.3% Triton-X-100, and 1% bovine serum albumin (BSA; Sigma-Aldrich, St. Louis, MO) in PBS. Sections were then incubated for 24 h at room temperature (RT) in a solution containing primary antibodies (PV: 1:10,000; CR: 1:1000 ChAT: 1:100; GFAP: 1:1000) in 1% normal non-immune serum, 0.3% Triton-X-100, and 1% BSA in PBS. On the following day and after PBS rinses, sections were incubated in a PBS solution containing (secondary) biotinylated horse anti-mouse IgGs (1:200 dilution; Vector Laboratories, Burlingame, CA) combined with 1% normal serum, 0.3% Triton-X-100, and 1% BSA for 90 min at RT. Sections were exposed to an avidin-biotin-peroxidase complex (ABC; 1:100 dilution, Vector Laboratories) for 90 min followed by rinses in PBS and Tris buffer (50 mM; pH 7.6). Sections were then incubated with a solution containing 0.025% 3, 3′-diaminobenzidine tetrahydrochloride (DAB; Sigma-Aldrich), 10 mM imidazole (Fisher Scientific, Pittsburgh, PA), and 0.006% hydrogen peroxide in Tris buffer for 10 minutes at RT. The sections were mounted on slides, dehydrated with increasing dilutions of ethanol followed by xylene, and then, the slides were coverslipped using Permount. A ScanScope light microscope (Aperio Technologies; Vista, CA) was used to image the immunostained sections. To test for possible regional differences in the extent of degeneration, we separated the serial sections into rostral and caudal striatal levels, using the anterior commissure as the landmark between the pre-commissural and post-commissural striatal regions (Supplemental Fig. 1).

### Stereology

The delineation of the regions of interest (ROI) was performed at 2.5x magnification, while cell counts were conducted with either a 40x oil-immersion objective for CR+, PV+ and ChAT+ interneurons counting or a 100x oil-immersion objective for Nissl-stained neurons counting. In Nissl-stained sections, only neuronal cell bodies with a clearly distinguishable nucleus were counted. These were easily distinguishable from glial cells based on the larger size of their somata.

The delineation of the caudate nucleus and putamen and the unbiased volume estimation were performed under low magnification (2.5x) by multiplying the sum of the caudate nucleus and putamen areas by the distance between the sections^22^. The ROI was divided and sampled in a superimposed virtual grid to maximize efficiency and minimize the coefficient of error (CE).The precision of the estimates of the total number of neurons was evaluated by the CE^23^ (Supplemental Table 2).

## Results

Because the two HD monkeys (rHD7, rHD1) used in this study were genotypically and phenotypically different in their expression of HD symptoms^3,4^, data from each animal are presented separately. For each of the three populations of striatal interneurons examined in these animals, both unbiased stereological counts and density values are discussed. Furthermore, in order to relate changes in the number of interneurons to the extent of the total neuronal loss in the striatum of the two HD monkeys, stereological counts of the total number of Nissl-stained neurons in the striatum of the control (rWT1) and the two HD animals (rHD7 and rHD1) are presented. Overall, the pattern of CR, PV, and ChAT immunoreactivity in the striatum of the control monkey was similar to that described in previous studies^18,24–26^.

### Total Number of Nissl-stained Neurons

**Figure 1:**
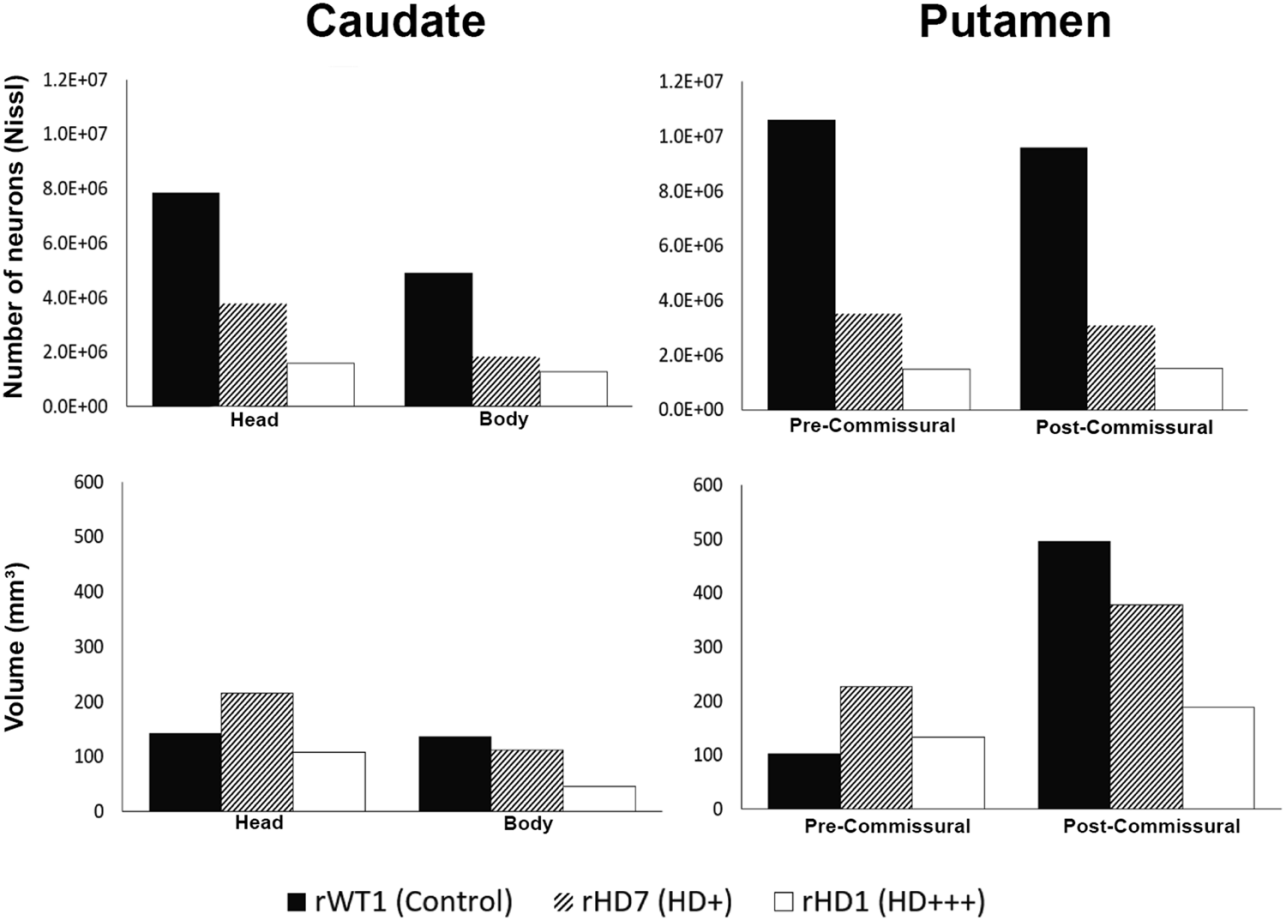
(**A**,**B**) Stereological counts of Nissl-stained neurons of the head and body of the caudate nucleus and in the pre- and post-commissural putamen in the two HD monkeys (rHD1, rHD7) and the control monkey (rWT1). (**C**,**D**) Volume calculations of the caudate nucleus and putamen from the Nissl-stained slices in the same animals. The CE for the volume calculations was at or below 0.02 for all measurement above.

Overall, the data presented in this section are consistent with those shown in our recent study^4^. The total number of neurons from Nissl-stained sections was decreased in the caudate nucleus and putamen of both HD monkeys when compared to rWT1. rHD1 displayed the most severe striatal cell loss, which ranged from nearly ~75% in the head and body of the caudate nucleus to ~85% in the pre- and post-commissural putamen. Albeit less pronounced, rHD7 also exhibited significant neuronal loss in the caudate head (52%) and body (63%) and in the pre-/post-commissural putamen (~67%) (Fig. 1A,B; Supplemental Table 3). Consistent with this massive neuronal loss, the volume of the caudate nucleus and putamen of rHD1 was reduced, ranging from 23% to 67% across the various striatal sections (Fig. 1C,D; Supplemental Table 4). The situation was different in rHD7. Despite the pronounced neuronal loss, the volume of the head of the caudate nucleus and pre-commissural putamen was larger than control (51% and 70%, respectively), while that of the caudate body and post-commissural putamen underwent 18% and 24% reduction, respectively, when compared to rWT1 (Fig. 1C,D; Supplemental Table 4).

### Total Number and Density of CR+ interneurons

**Figure 2:**
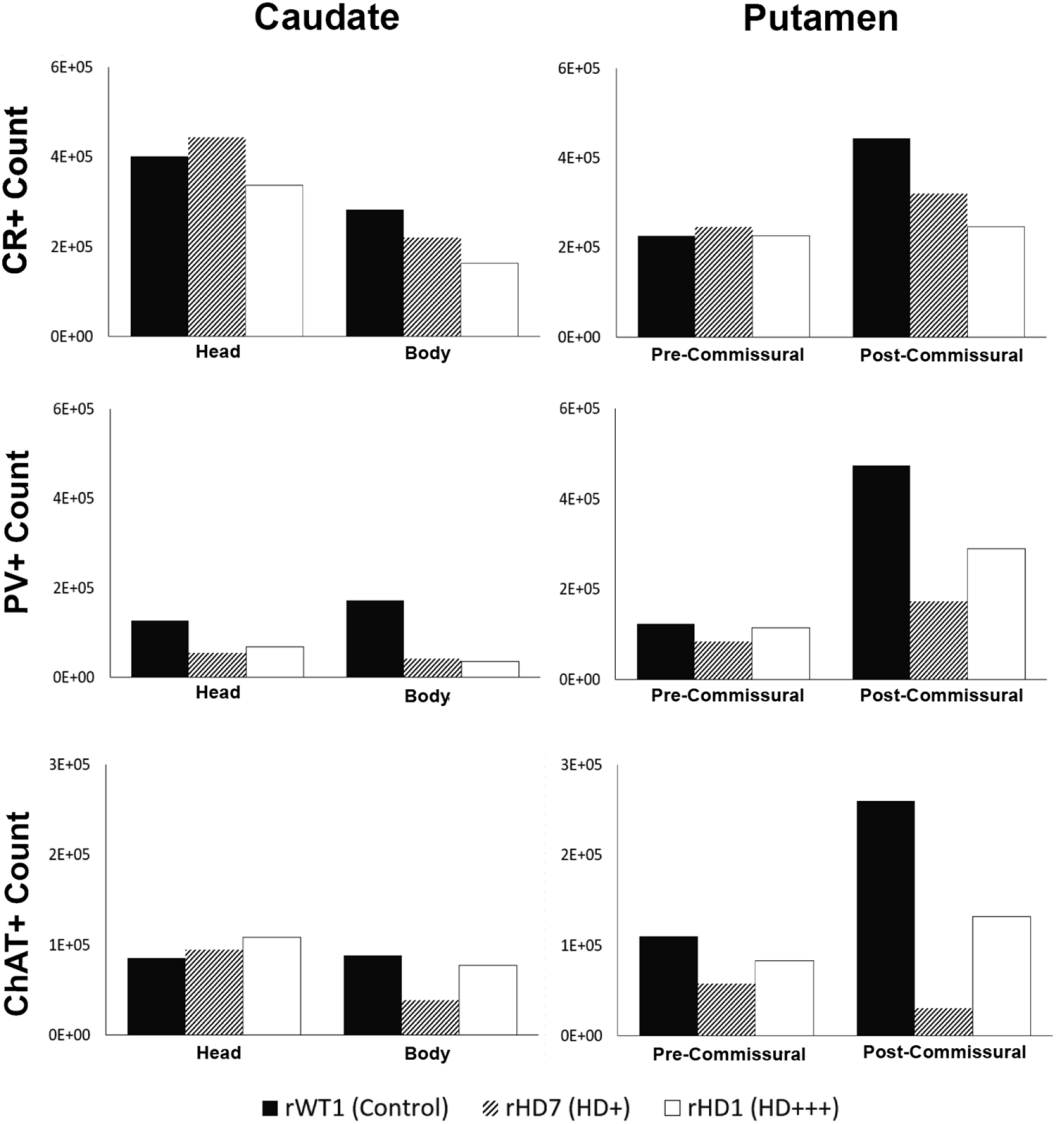
(**A**–**F**) Estimated total number of CR+ cells (**A**,**B**), PV+ cells (**C**,**D**) and ChAT+ cells (**E**,**F**) in the caudate nucleus and putamen of the two HD monkeys (rHD1, rHD7) and the control animal (rWT1). The CE was at or below 0.08 for all measurements shown above.

Monkey rHD1 had 16% and 42% fewer CR+ neurons in the caudate head and body, respectively, than rWT1. rHD7 was less affected, exhibiting a 22.1% loss in the caudate body, but a slight gain (~10%) in the caudate head compared to the control. The pre-commissural putamen of both rHD1 and rHD7 did not display any major change in the number of CR+ neurons, but the post-commissural putamen exhibited 44% and 25% CR+ neuronal loss in rHD1 and rHD7, respectively (Fig. 2A,B; Supplemental Table 3).

**Figure 3:**
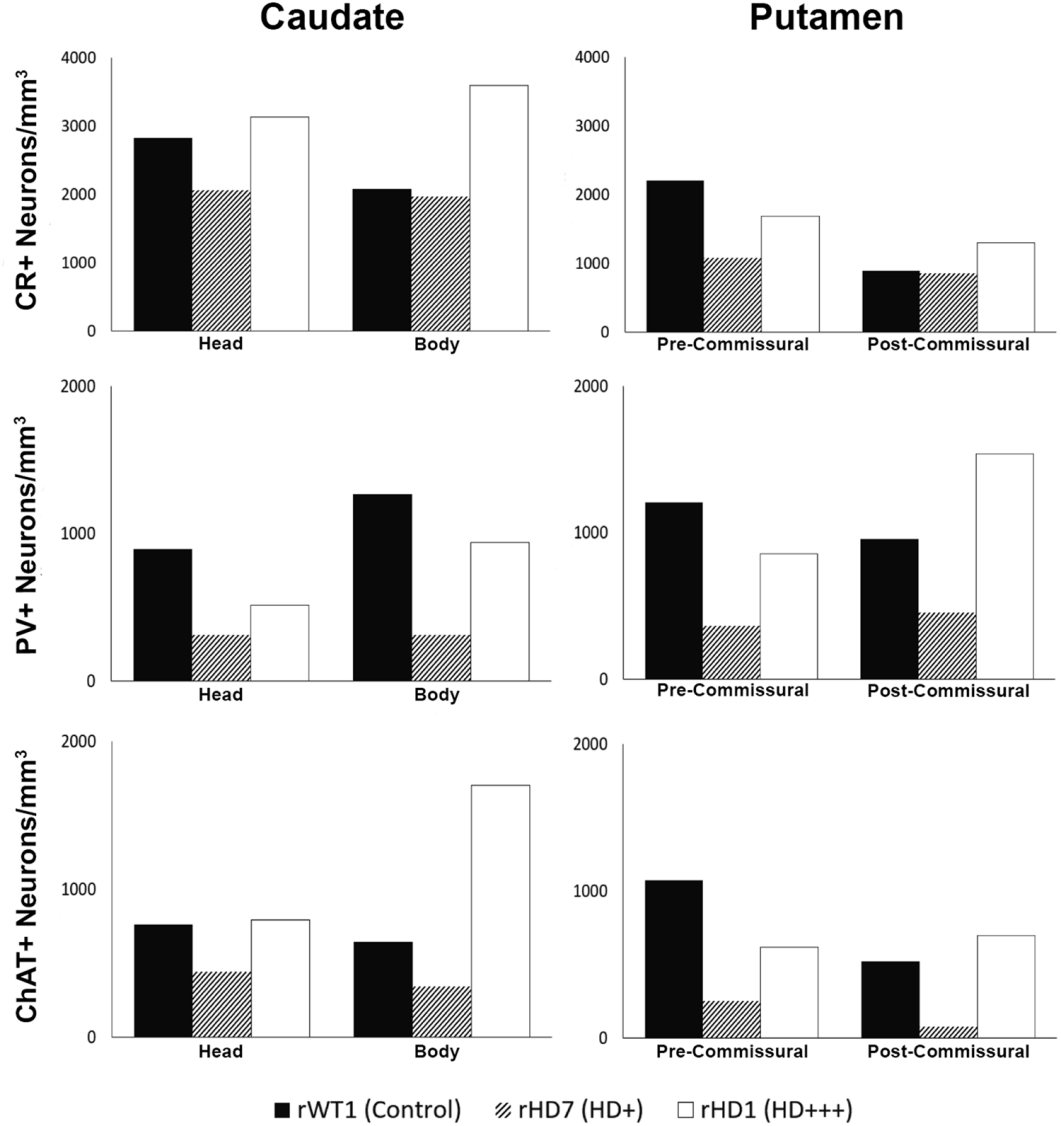
(**A**–**F**) Estimated density of CR+ cells (**A**,**B**), PV+ cells (**C**,**D**), and ChAT+ cells (**E**,**F**) in the caudate nucleus and putamen of the two HD monkeys (rHD1, rHD7) and the control animal (rWT1).

**Figure 4:**
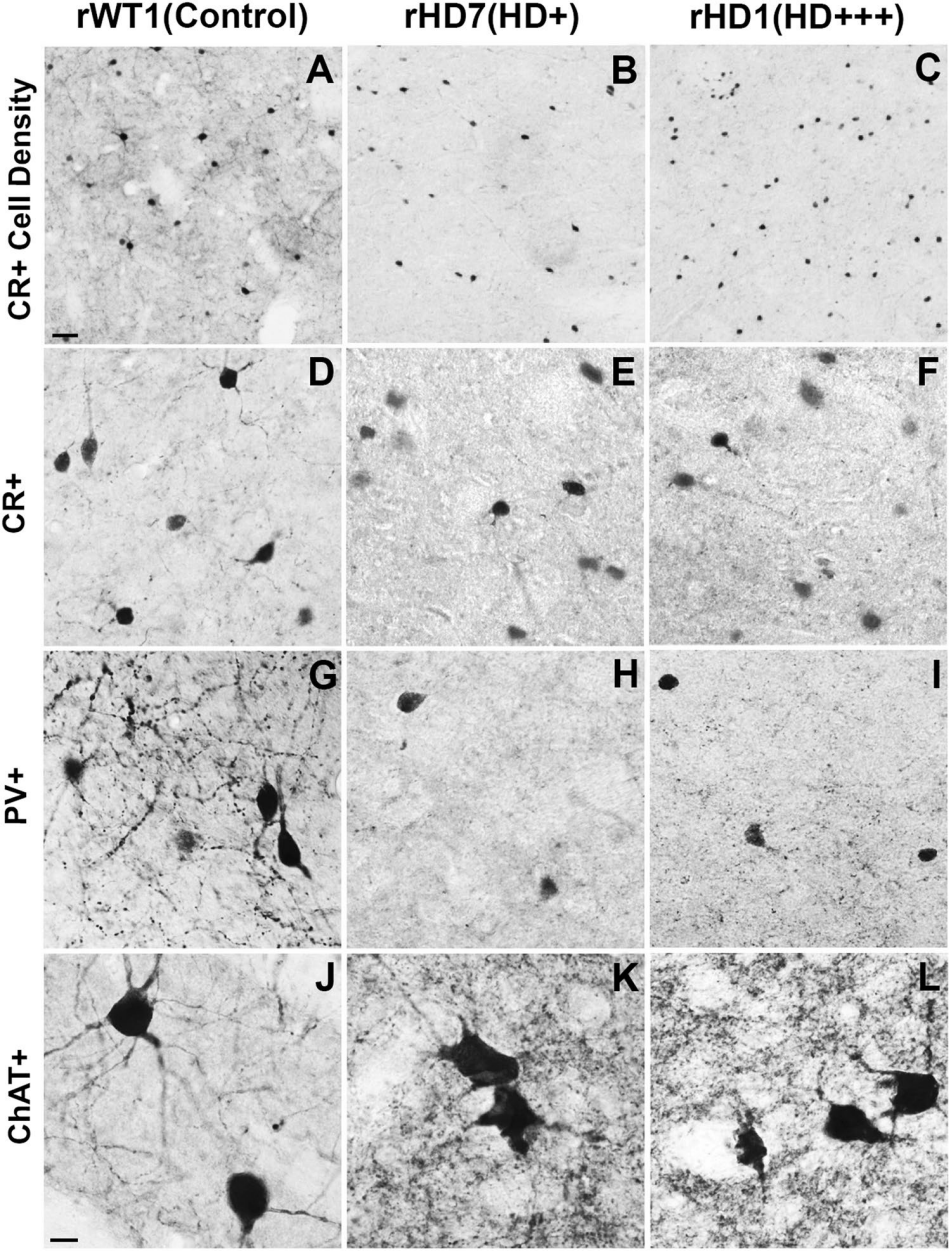
(**A**–**C**) Light microscope images depicting the differences in density of CR+ cells in the caudate head between the two transgenic HD (rHD1, rHD7) and WT (rWT1) monkeys. The scale bar in A represents 5 μm. (**D**–**L**) Light microscope images depicting examples of striatal CR+, PV+, and ChAT+ neurons in each monkey. The scale bar in J represents 10 μm.

To quantify the density of CR+ interneurons (and other interneuron subtypes), the total number of neurons counted in each of the four striatal sub-regions was divided by the volume of these corresponding areas measured using the Cavalieri’s principle from Nissl-stained sections. Taking these volume measurements in consideration, rHD1 displayed an increase in the density of CR+ neurons throughout all striatal regions, except in the pre-commissural putamen. Unlike rHD1, rHD7 showed no change in CR+ neurons density in the body of the caudate nucleus and the post-commissural putamen, while it displayed a decreased density (27–51%) in the head of the caudate nucleus and pre-commissural putamen (Figs 3A,B and 4; Supplemental Table 5).

### Total Number and Density of PV+ Neurons

Both HD monkeys displayed a major loss of PV+ neurons in the caudate nucleus compared to control. rHD1 had 46% and 79% fewer PV+ cells in the caudate head and body, respectively, while the same regions contained 56% and 75% fewer PV+ neurons than controls in rHD7. A decrease in PV+ neurons was also found in the pre- and post-commissural putamen of rHD1 (8–40%) compared to rWT1, albeit it was not as severe as in rHD7, which had as much as 60–80% lower PV+ density in caudal striatal regions (Figs 2C,D and 4; Supplemental Table 3).

For PV+ density analysis, both HD monkeys showed decrease in PV+ cell density across all regions of the striatum, except for the post-commissural putamen in rHD1 which had a 60% greater PV+ density when compared to rWT1 (Fig. 3C,D; Supplemental Table 5).

### Total Number and density of ChAT-positive Neurons

All striatal regions analyzed displayed a variable degree of ChAT+ cell loss in rHD1 and rHD7, except for the head of the caudate nucleus, which contained a slightly larger number (+27% and +11% in rHD1 and rHD7, respectively) of ChAT+ neurons than in rWT1. In contrast, the body of the caudate nucleus displayed a 12% and 56% ChAT+ cell loss in rHD1 and rHD7, respectively. In the pre- and post-commissural putamen, the number of ChAT+ neurons was lower in both HD monkeys than in control. As was the case for the body of the caudate nucleus, the extent of cell loss was more pronounced in rHD7 than in rHD1. The percent ChAT+ neurons loss ranged between 48–88% in the pre- and post-commissural putamen of rHD7, while the same striatal regions displayed 25–49% neuronal loss in rHD1 (Fig. 2E,F; Supplemental Table 3).

The density of ChAT+ neurons was increased in all striatal regions of monkey rHD1 (+4% to +164%), except in the pre-commissural putamen. In contrast, rHD7 showed reductions in ChAT+ density (42–85%) across all striatal regions (Figs 3E and 4; Supplemental Table 5).

### Relative Sparing of Striatal Interneurons in HD Monkeys

In light of human data suggesting a relative sparing of striatal interneurons in HD^24^, we determined whether such was also the case in HD monkeys by comparing the percent of each interneuron population relative to the total number of neurons in the striatum of the control and the two HD monkeys. CR+ interneurons comprised 4% of total neurons in the striatum of rWT1, while this percentage increased to 17% and 10% in rHD1 and rHD7, respectively. Similarly, while PV+ interneurons encompassed 3% of total striatal neurons in rWT1, this percentage was 9% and 3% in rHD1 and rHD7, respectively. Along the same lines, ChAT+ interneurons accounted for 2% of the total striatal population in rWT1, while they comprised 7% and 2% of total striatal neurons in rHD1 and rHD7, respectively. Overall, these data suggest that the rate of striatal interneurons loss in HD monkeys is not as pronounced as that of the general neuronal population (Fig. 4).

### Striatal Astrogliosis in HD Monkeys

**Figure 5:**
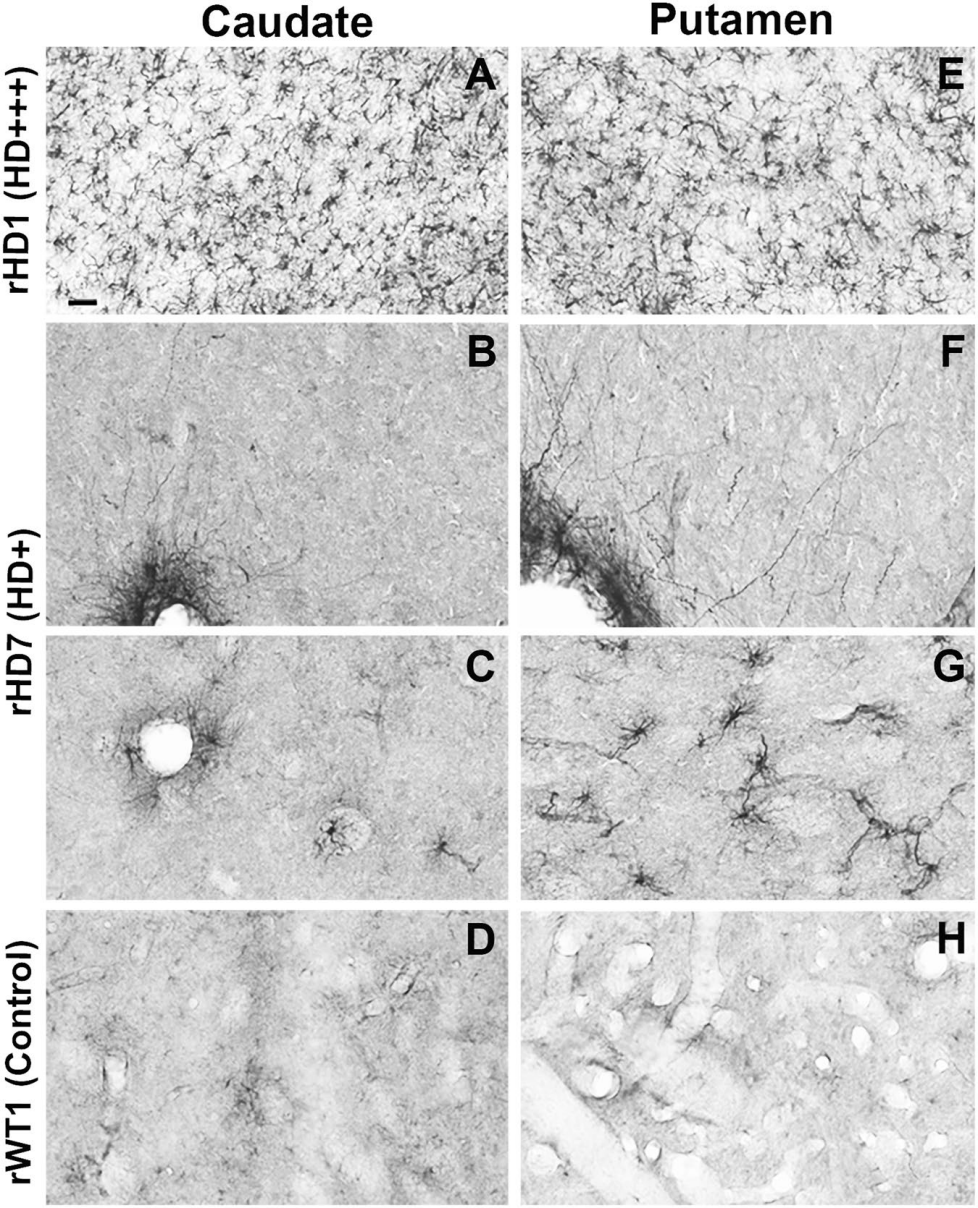
(**A**–**H**) Light micrographs showing astrocytic GFAP labeling in the caudate nucleus (CD; **A**–**D**) and putamen (PU; **E**–**H**) of the two transgenic HD monkeys (rHD1, rHD7) and the control animal (rWT1). Note the large density of labeled astrocytes in the CD and PU of the most severely affected HD monkey (rHD1-**A**,**E**) compared with the least affected HD monkey (rHD7; **B**,**C**; **F**–**G**) and the control (**D**,**H**). In the CD of rHD7, GFAP-immunostained elements were confined to the membrane of blood vessels (**B**), with very rare instances of labeled neuropil astrocytes (**C**), while in the PU, a modest number of immunoreactive astrocytes could be seen, predominantly in the ventral tier of the structure (**G**). Scale bar in A: 30 um in A and E; 20 um in (**B**–**D** and **F**–**H**).

As depicted in Fig. 5, both the caudate nucleus and putamen of rHD1 were invaded by a profuse meshwork of GFAP-positive astrocytes (Fig. 5A,E), while the striatal neuropil of rHD7 was almost completely devoid of labeled astrocytes, except for a sparse population in the ventral part of the putamen (Fig. 5B,C,F,G). Additionally, strongly GFAP-labeled astrocytic processes were found along the external membrane of blood vessels throughout the striatum of this animal (Fig. 5B,F). In contrast to the two HD animals, the striatum of the control monkey (rWT1) was devoid of GFAP-immunoreactive elements (Fig. 5D,H).

## Discussion

The results of this study further increase the face validity of the first transgenic rhesus monkey model of HD^7^. Combined with the evidence for the progressive development of cognitive and motor impairments reported in these monkeys^3,4^, the present data demonstrate that striatal projection neurons and interneurons undergo pathological changes that closely resemble those reported in HD patients^10,11,24–27^. As expected on the basis of their differential genotype and severity of their symptoms, the reduction in striatal volume, which reached as much as 50–75% throughout the whole striatum in rHD1, was much less pronounced in rHD7. As shown in HD patients^10^, there was a caudo-rostral gradient in the extent of striatal degeneration in both transgenic monkeys, i.e. the posterior regions of the caudate nucleus and putamen displayed a more severe volume reduction than anterior striatal regions. Furthermore, our findings provide strong evidence for degeneration of both projection neurons and interneurons in HD monkeys, albeit to variable degrees, with PV+ GABAergic interneurons undergoing the most severe degeneration. Our findings about the differential increased expression of GFAP-positive reactive astrocytes in the most severe HD animal (rHD1) compared with the less affected monkey (rHD7) is also consistent with previous human postmortem studies^10,11^.

At first glance, the loss of striatal interneurons seen in HD monkeys may appear at odds with the large amount of human literature suggesting a relative sparing of striatal interneurons in HD^10,24–27^. However, it is noteworthy that these conclusions were largely based on interneurons density measurements. Because the striatal volume of advanced HD patients is dramatically reduced^10–12^, these cellular density assessments must be interpreted with caution, and do not provide information about the absolute number of striatal interneurons in HD. In fact, our cell density measurements for all populations of interneurons in the most affected HD monkey (rHD1) are consistent with the human literature and support the suggestion that most striatal interneurons do not degenerate at the same pace as projection neurons in HD.

However, there are some important limitations that need to be taken into consideration in the interpretation of our findings. First and foremost is the low number of animals. As additional HD monkeys become available, future studies should be undertaken to validate and extend our preliminary observations. For instance, although preliminary data from the current HD monkeys suggest a decreasing severity of the degenerative process along the medio-lateral and the dorso-ventral axis of the rostral striatum, as reported in HD patients^10,11^, studies in a larger cohort of animals are needed to confirm these findings. Second, our data does not allow us to make a firm statement that the decreased numbers of interneurons reported in this study resulted from a genuine neuronal degeneration of specific interneuron subtypes or a decrease in the level of proteins targeted by the antibodies (i.e. CR, PV or ChAT) in each neuronal subtype. Although both changes are important, and may have critical functional consequences in striatal processing, the use of additional markers to label some of these neuron subtypes in a larger cohort of HD animals should help sort out this issue. The possibility for fluctuating changes in protein expression during disease progression could explain some of our data showing a larger number of PV+ or ChAT+ interneurons in the post-commissural putamen of rHD7 than rHD1.

Despite the fact that lingering issues remain to be addressed in larger cohorts of HD monkeys, our pathological data, together with the imaging and behavioral observations collected from the two monkeys used in this study^3,4^, further validate our HD monkey model in recapitulating some key aspects of the striatal neuropathology and behavioral symptoms of human HD. These data provide a solid foundation for the continued development and characterization of the HD monkey model as a valuable tool for studying HD pathogenesis and for developing novel treatment strategies.

## Supplementary information

Supplementary Information

## Data Availability

Once accepted for publication all data presented in this manuscript will be made available to anyone interested
