# Supplementary material for: Striatal Interneurons in Transgenic Nonhuman Primate Model of Huntington’s Disease
Source: Sci Rep. 2019 Mar 5;9:3528. doi: 10.1038/s41598-019-40165-w (PMC6401084; doi:10.1038/s41598-019-40165-w)
Supplement: Supplementary file 1 — Supplementary Information [file 41598_2019_40165_MOESM1_ESM.pdf]

Striatal Interneurons in Transgenic Nonhuman Primate Model of Huntington's Disease

Shoeb B. Lallani[1,3], Rosa M. Villalba[3.4], Yiju Chen[1,3], Yoland Smith[2,3,4]*, Anthony W.S. Chan[1,3]*

[1]Department of Human Genetics and [2]Department of Neurology, Emory University School of Medicine, [3]Division of Neuropharmacology and Neurological Diseases, Yerkes National Primate Research Center, Atlanta, GA, USA, [4]UDALL Center for Excellence for Parkinson's Disease, Emory University, Atlanta, GA, USA

SUPPLEMENTARY MATERIAL

Supplementary Material

		CR+		PV+		ChAT+	
		Number of sections	*Interval Number*	*Number of sections*	*Interval Number*	*Number of sections*	*Interval Number*
**rWT1**	Pre-Commissural	8	1/15	8	1/15	6	1/24
	Post-Commissural	16	1/15	16	1/15	11	1/24
**rHD7**	Pre-Commissural	6	1/24	5	1/24	15	1/12
	Post-Commissural	8	1/24	7	1/24	19	1/12
**rHD1**	Pre-Commissural	5	1/24	4	1/24	9	1/12
	Post-Commissural	7	1/24	5	1/24	13	1/12

Supplemental Table 1. Number of Sections and Serial Section Interval Number
The number of sections that were stained with each immunomarker and the serial section interval number for each immunomarker and NHP.

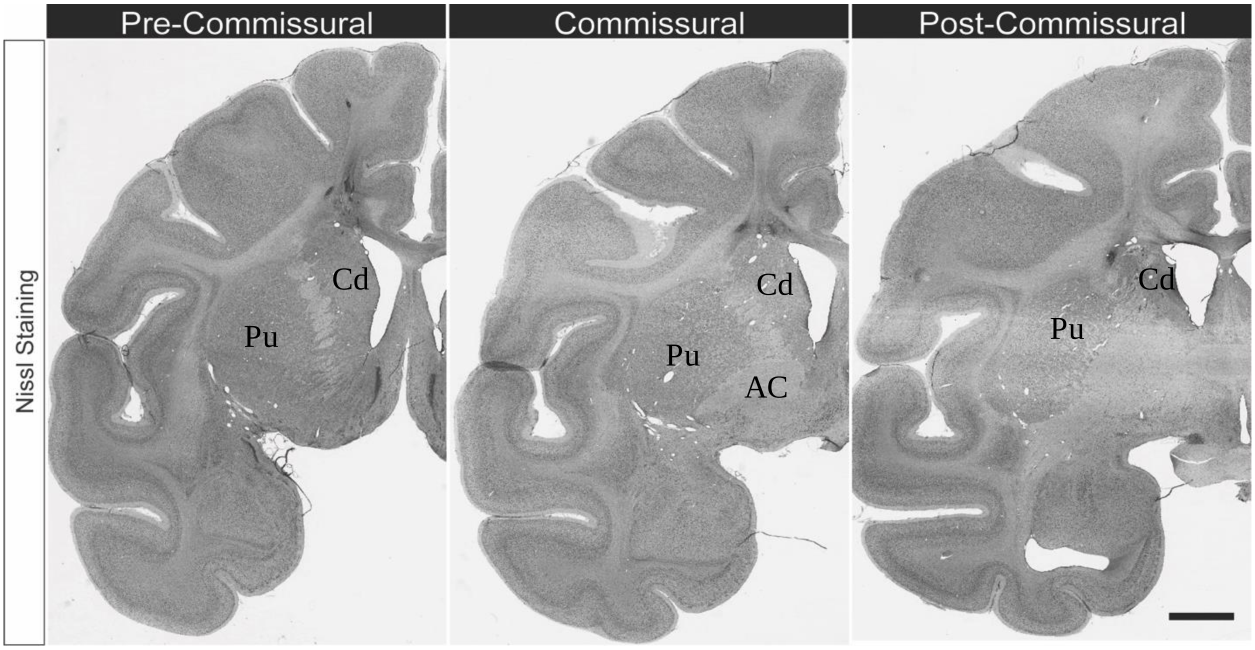

Supplemental Figure 1. Pre- and Post-Commissural Regions

Scanned images of Nissl-stained coronal brain sections (50µm) depicting the striatal ROIs – the caudate nucleus (Cd) and putamen (Pu). The middle micrograph shows the anterior commissure (AC), which is used as the landmark that distinguishes the head (anterior to AC) from the body (posterior to AC) of the caudate nucleus, and the pre- from the post-commissural regions of the putamen. The scale bar represents 5mm.

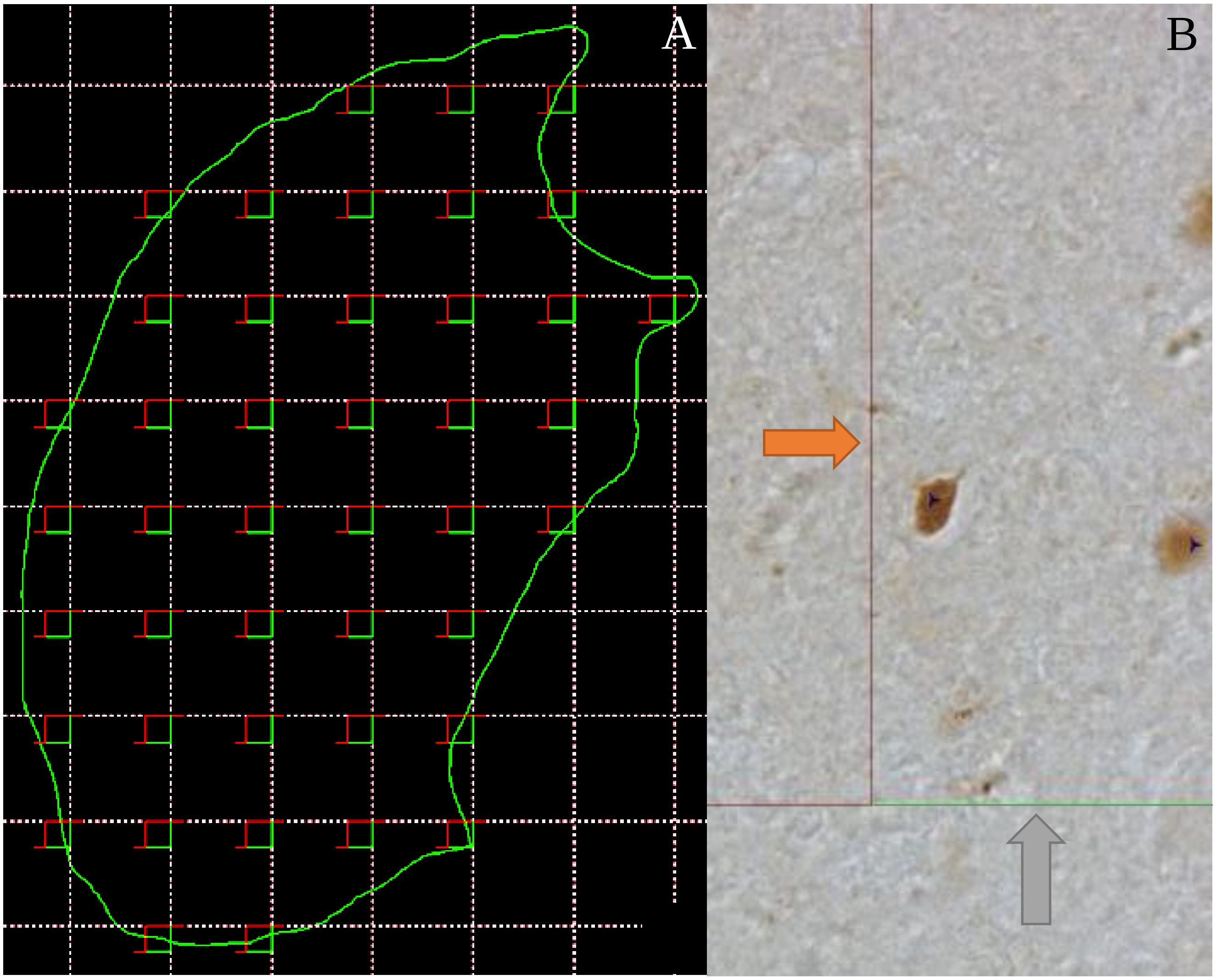

Supplemental Figure 2. Stereology

(A) Optical fractionator grid (white lines) and counting probes (red and green squares) at 2.5x. The ROI is traced in green. (B) The stereology probe under 40x oil-immersion. Depicted are the acceptance (green) and rejection (red) lines.

Target	Grid/Probe	Caudate Nucleus	Putamen
CR	Grid	700 µm x 700 µm	1200 µm x 1200 µm
	Probe	300 µm x 300 µm	300 µm x 300 µm
PV	Grid	700 µm x 700 µm	800 µm x 800 µm
	Probe	300 µm x 300 µm	400 µm x 400 µm
ChAT	Grid	1400 µm x 1400 µm	1400 µm x 1400 µm
	Probe	600 µm x 600 µm	600 µm x 600 µm

Supplemental Table 2. Optical Fractionator Grid and Counting Probe Size
The optical fractionator grid and counting probe size. The dimensions were chosen to maximize efficiency and minimize the CE.

	**Caudate Nucleus**				**Putamen**			
	Head		*Body*		*Pre-Commissural*		*Post-Commissural*	
	Estimated Total Cells	Percent Difference from rWT1	Estimated Total Cells	Percent Difference from rWT1	Estimated Total Cells	Percent Difference from rWT1	Estimated Total Cells	Percent Difference from rWT1
Nissl+ cells								
rWT1	7.85E+06	-	4.92E+06	-	1.06E+07	-	9.58E+06	-
rHD7	3.77E+06	-52.0%	1.84E+06	-62.6%	3.51E+06	-67.0%	3.09E+06	-67.7%
rHD1	1.60E+06	-80.0%	1.28E+06	-74.0%	1.48E+06	-86.1%	1.50E+06	-84.3%
CR+ cells								
rWT1	4.01E+05	-	2.83E+05	-	2.26E+05	-	4.43E+05	-
rHD7	4.43E+05	+10.5%	2.21E+05	-21.9%	2.45E+05	+8.4%	3.30E+05	-25.5%
rHD1	3.35E+05	-16.5%	1.63E+05	-42.4%	2.26E+05	0%	2.46E+05	-44.5%
PV+ cells								
rWT1	1.27E+05	-	1.72E+05	-	1.24E+05	-	4.74E+05	-
rHD7	6.79E+04	-56.3%	3.50E+04	-75.2%	8.37E+04	-32.5%	1.72E+05	-63.7%
rHD1	5.55E+04	-46.5%	4.27E+04	-79.7%	1.14E+05	-8.1%	2.90E+05	-38.8%
ChAT+ cells								
rWT1	8.52E+04	-	8.78E+04	-	1.10E+05	-	2.60E+05	-
rHD7	9.49E+04	+11.4%	3.84E+04	-56.3%	5.75E+04	-47.7%	3.01E+04	-88.4%
rHD1	1.08E+05	+26.8%	7.73E+04	-11.9%	8.31E+04	-24.5%	1.32E+05	-49.2%

Supplemental Table 3. Striatal Cell Counts and Percent Difference
Estimated total stereological counts for each interneuron and percent difference from rWT1 (control). Negative percentages indicate a lower number relative to the control, while a positive percentage indicates a higher number relative to the control.

	**Caudate Nucleus**				**Putamen**			
	Head		*Body*		*Pre-Commissural*		*Post-Commissural*	
	Estimated Total Volume (mm^3)	Percent Difference from rWT1	Estimated Total Volume (mm^3)	Percent Difference from rWT1	Estimated Total Volume (mm^3)	Percent Difference from rWT1	Estimated Total Volume (mm^3)	Percent Difference from rWT1
rWT1	142.0	-	136.1	-	133.6	-	495.6	-
rHD7	214.9	+51.4%	112.1	-17.6%	226.8	+69.8%	377.4	-23.9%
rHD1	107.4	-24.3%	45.3	-66.7%	102.6	-23.2%	188.6	-61.9%

Table 4. Striatal Volume and Percent Difference
Estimated striatal volume determined for the Nissl-stained sections and percent difference from rWT1 (control). Negative percentages indicate a lower volume relative to the control, while a positive percentage indicates a higher volume relative to the control.

	**Caudate Nucleus**				**Putamen**			
	Head		*Body*		*Pre-Commissural*		*Post-Commissural*	
	Estimated Total Density (n/mm^3)	Percent Difference from rWT1	Estimated Total Density (n/mm^3)	Percent Difference from rWT1	Estimated Total Density (n/mm^3)	Percent Difference from rWT1	Estimated Total Density (n/mm^3)	Percent Difference from rWT1
CR+ density								
rWT1	2.83E+03	-	2.08E+03	-	2.20E+03	-	8.94E+02	-
rHD7	2.06E+03	-27.1%	1.97E+03	-5.4%	1.08E+03	-50.9%	8.49E+02	-5.1%
rHD1	3.14E+03	+10.9%	3.60E+03	+73.1%	1.69E+03	-23.0%	1.31E+03	+45.8%
PV+ density								
rWT1	8.95E+02	-	1.27E+03	-	1.21E+03	-	9.57E+02	-
rHD7	3.16E+02	-64.7%	3.14E+02	-75.2%	3.69E+02	-69.4%	4.57E+02	-52.2%
rHD1	5.16E+02	-42.4%	9.42E+02	-25.6%	8.57E+02	-29.0%	1.54E+03	+60.6%
ChAT+ density								
rWT1	7.62E+02	-	6.45E+02	-	1.07E+03	-	5.25E+02	-
rHD7	4.41E+02	-42.1%	3.42E+02	-47.0%	2.53E+02	-76.4%	7.98E+01	-84.8%
rHD1	7.92E+02	+4.0%	1.71E+03	+164.4%	6.22E+02	-42.0%	6.98E+02	+33.1%

Supplemental Table 5. Striatal Density and Percent Difference
Density of each interneuron type and percent difference from rWT1 (control). Negative percentages indicate a lower number relative to the control, while a positive percentage indicates a higher number relative to the control. n = estimated number of neurons.
